# A computational and behavioral study of the precision of visuo-spatial working-memory for several items

**DOI:** 10.1186/1471-2202-12-S1-P45

**Published:** 2011-07-18

**Authors:** Rita Almeida, Albert Compte

**Affiliations:** 1 Institut d'Investigacions Biomèdiques August Pi i Sunyer (IDIBAPS), Barcelona, Spain

## 

A putative neuronal correlate of working-memory (WM) is the so called persistent or delay activity, that is the stimulus selective elevated neuronal firing observed long after stimulus offset. Neuronal activity with these characteristics has been found in several cortical areas in association with the performance of visuo-spatial working-memory (vsWM) tasks. One proposed computational model accounts both for electrophysiological measurements from monkeys [1] and for behavior and neuroimaging measurements from humans [2] acquired during a vsWM task that requires memorizing positions located on a circle. This computational model consists of a network of integrate-and-fire excitatory and inhibitory neurons organized according to a ring topography in terms of internal connectivity and external inputs received. The topography enables the model to sustain a line attractor mechanism for vsWM. This assumption of a continuum vsWM is essential for the model but has never been unequivocally demonstrated experimentally. Here, we address this issue combining computational modeling and behavioral experiments.

In the model the selectivity of the neurons is determined by the external inputs, which encode locations on a circle and hence can be completely characterized by angles. Upon stimulus presentation a subset of neurons, whose selectivities are centered on the angle of the stimulus, shows elevated firing activity that persists after stimulus offset. When several items are presented at the same time several subsets of neurons will show elevated activity corresponding to a memory trace for each item. However, due to the ring structure of the model, the stability of the different memory traces will depend on their relative positions. If two items are located in nearby locations the corresponding memory traces will interfere and they will merge with high probability. So, the hypothesis that there is a topographic structure in the circuits supporting vsWM leads to the prediction that the efficiency with which different items are memorized depends on their relative locations. In this work we tested this prediction using behavioral experiments in humans. The experimental results were consistent with the theoretical prediction. In particular, we found evidence consistent with the predicted merging of memory traces for nearby locations.

The effect described can also be at the origin of recent experimental results [3] showing that there is a decrease of precision of vsWM with the increase of number of items to be memorized. These results were used to challenge the traditional way in which WM ability has been characterized in terms of capacity, that is in terms of the number of items one can keep in mind at the same time. WM capacity has been thought to be limited to a fixed number of items, while these recent results suggest that one should think of WM in terms of a pool of limited resources that is shared among the items to be memorized. The results from our computational study suggest mechanisms at the neuronal population level which can account for the experimental evidence supporting the two opposing views of working memory capacity.
